# Field Studies on the *Saccharomyces* Yeast Strain MIIP as an Immunomodulator to Mitigate PRRSV Infection in Piglets

**DOI:** 10.3390/vetsci13020175

**Published:** 2026-02-10

**Authors:** Wei-Ting Lin, Mao-Yuan Du, Ishin Tseng, Ming-Tang Chiou, Hsiang-Jung Tsai, Chao-Nan Lin

**Affiliations:** 1 Department of Veterinary Medicine, College of Veterinary Medicine, National Pingtung University of Science and Technology, Pingtung 91201, Taiwan; winnie2001900125@gmail.com (W.-T.L.); abc972401@gmail.com (M.-Y.D.); mtchiou@mail.npust.edu.tw (M.-T.C.); 2 Institute of Epidemiology and Preventive Medicine, College of Public Health, National Taiwan University, Taipei 106319, Taiwan; d12849007@ntu.edu.tw; 3 Animal Disease Diagnostic Center, College of Veterinary Medicine, National Pingtung University of Science and Technology, Pingtung 91201, Taiwan; 4 Research and Technical Center for Sustainable and Intelligent Swine Production, National Pingtung University of Science and Technology, Pingtung 91201, Taiwan; 5 School of Veterinary Medicine, National Taiwan University, Taipei 106319, Taiwan

**Keywords:** PRRSV (porcine reproductive and respiratory syndrome virus), *Saccharomyces cerevisiae* MIIP, immunomodulation, mucosal immunity, swine health management

## Abstract

Porcine reproductive and respiratory syndrome virus (PRRSV) remains a significant threat to swine health worldwide, primarily due to its extensive genetic diversity and ability to evade host immune responses, which reduces the effectiveness of vaccines. This field study investigated the immunomodulatory effects of the *Saccharomyces cerevisiae* strain MIIP as a dietary supplement to mitigate PRRSV infection in piglets under commercial farming conditions. Four trials were conducted across PRRSV-affected herds, with variations in PRRS vaccination practices and infection status. MIIP supplementation led to a significant reduction in the PRRSV antibody-positive rate, titers, and viremia in treated piglets compared to both non-concurrent and concurrent control groups. Enhanced mucosal immunity was also observed, as evidenced by higher total IgA concentrations in serum and milk in the MIIP groups. Although not all immune parameters reached statistical significance, trends consistently favored improved antiviral responses in MIIP-treated animals. These results suggest that dietary supplementation with MIIP yeast can enhance both systemic and mucosal immunity, potentially reducing PRRSV infection and transmission. Further research into its mechanisms, particularly its interaction with innate immune pathways and the gut microbiota, is warranted to optimize PRRSV control strategies.

## Simple Summary

This field study on pig farms in Taiwan aimed to evaluate whether supplementing pig diets with MIIP yeast (a strain of *Saccharomyces cerevisiae*) could help prevent or reduce infection with porcine reproductive and respiratory syndrome virus (PRRSV). The results show that pigs receiving the yeast supplement had lower levels of the virus and antibodies in their blood, indicating reduced infection. On farms where piglets are typically infected between 3 and 6 weeks of age, most piglets in the MIIP group remained uninfected, while those in the control group showed 100% infection. The supplement also enhanced immune function by raising IgA levels in the blood and milk of both piglets and sows. However, once supplementation stopped, infection rates rose again, indicating that continuous use is needed. Overall, MIIP yeast is a promising tool to improve pig health and immunity against PRRSV.

## 1. Introduction

Porcine reproductive and respiratory syndrome (PRRS) is a significant challenge disease in global swine production, leading to reproductive failure and respiratory distress, with substantial economic implications. The causative agent, porcine reproductive and respiratory syndrome virus (PRRSV), is classified into two distinct species: *Betaarterivirus suid 1* (PRRSV-1, previously referred to as the European type) and *Betaarterivirus suid 2* (PRRSV-2, formerly known as the North American type) [1]. PRRSV-1 and PRRSV-2 have both been identified in Taiwan; however, PRRSV-2 remains the dominant strain and is associated with significant economic losses in the nursery stage [2,3].

PRRSV exhibits extensive genetic diversity and immunological complexity, posing significant challenges to its eradication. The virus’s persistence as a global concern is largely attributed to the rapid induction of non-neutralizing antibodies, together with a delayed appearance of neutralizing antibodies, typically emerging around 3-4 weeks post-infection [4,5,6] or vaccination [7]. This humoral immune response imbalance is further exacerbated by the delayed development of a virus-specific cell-mediated immune response, characterized by an insufficient innate interferon and cytokine response during PRRSV infection [4,8,9,10]. In addition to immunological challenges, the extensive genetic variability of PRRSV, alongside its frequent recombination events, significantly compromises vaccine efficacy [11,12,13,14,15,16,17,18,19]. These genomic alterations contribute to antigenic drift and shift, hindering the development of broadly protective vaccines and complicating long-term disease control strategies. Further investigating the molecular mechanisms underlying PRRSV immune evasion, recombination dynamics are crucial for improving vaccine design and enhancing protective immunity against diverse viral strains. In addition, an integrated approach that targets the enhancement of the innate and/or adaptive immune responses may represent a pathway toward more effective disease mitigation.

*Saccharomyces* yeast has been recognized as a resilient probiotic with a strong capacity to withstand gastrointestinal stress, exhibiting high survival rates in the stomach and small intestine. *Saccharomyces* yeast has been shown to enhance innate immunity and strengthen both local and systemic immune responses. Its effects are particularly evident in the induction of IgA production, which has been well documented [20,21,22,23,24]. It is presumed that oral administration of *Saccharomyces* yeast has immunomodulatory roles via enteric mucosal immune responses, including both innate and adaptive defense mechanisms to prevent microbial infections and maintain intestinal homeostasis [25]. These mechanisms may involve internalization by dendritic cells (DCs) and macrophages, thereby facilitating antigen presentation and contributing to immune system activation [26]. Additionally, yeast supplementation has been shown to increase leukocyte populations, IgG concentrations, and PRRSV-specific antibody responses, including T-helper (Th) and cytotoxic T (Tc) cell activity in piglets receiving PRRSV vaccinations [27].

This study investigates the efficacy of brewer’s yeast strain MIIP in preventing and treating PRRS under field conditions, exploring its potential as an immune-boosting dietary supplement that can regulate host defense mechanisms against PRRSV infection.

## 2. Materials and Methods

### 2.1. Yeast Product

The preparations of MIIP live yeast *Saccharomyces cerevisiae* (strain MIIP/ST01) were obtained by high-cell-density fermentation in a 5000 L fermenter (GeneFerm Biotechnology Co., Ltd., Tainan, Taiwan). The fermentation liquor was freeze-dried to produce 1 × 10^7^ CFU/g viable yeast cells. Inactivated yeast was prepared by using 10–30 kGy of gamma radiation for 1 h (China Biotech Corporation, Taichung, Taiwan).

### 2.2. PRRSV ELISA and Total IgA Quantification

PRRSV antibody levels in serum were measured using a commercial enzyme-linked immunosorbent assay (ELISA) kit (IDEXX PRRS X3 Ab Test, IDEXX Laboratories, Inc., Westbrook, ME, USA). All operating procedures were carried out according to the commercial kit protocol. PRRSV-specific antibody titers were reported as sample-to-positive (S/P) ratios, with a value of 0.4 or higher considered positive. For total pig IgA quantification, ELISA kits (Bethyl Laboratory, Inc., Montgomery, TX, USA) were used according to the manufacturer’s protocol.

### 2.3. PRRSV qPCR

Total nucleic acids were extracted from serum samples using a MagNA Pure LC total nucleic acid isolation kit (Roche Diagnostics GmbH, Mannheim, Germany) operated on a MagNA Pure 96 System (Roche Applied Science, Rotkreuz, Switzerland) according to the manufacturer’s protocol. All samples were tested for PRRSV using a quantitative real-time polymerase chain reaction (qPCR) [28].

### 2.4. Viral Neutralization Assay

The viral neutralization assay was performed in MARC145 cells (American Type Culture Collection, ATCC No. CRL-12231) using PRRSV-2 strain 763 (GenBank accession no. KY073240) against two-fold diluted milk samples. An immunofluorescence assay (IFA) was used to identify cytopathic effects (CPEs), and the neutralizing titers were determined as the highest dilutions without CPEs [29].

### 2.5. Experimental Design

**Trial 1.** The field trial was conducted in a farrow-to-finish herd with 150 sows in Taiwan. This herd had a documented history of PRRS outbreaks, with no PRRS vaccination implemented on the farm. Over five months, all batches of nursery piglets in the herd were fed a diet supplemented with 0.1% MIIP from weaning at four weeks of age until 12 weeks of age. Serum samples were randomly collected from five piglets, each eight weeks old at the time of sampling, at the initiation of the trial, upon cessation of MIIP supplementation, and two months after treatment discontinuation. These samples were analyzed for PRRS ELISA antibody levels to monitor immune response dynamics.

**Trial 2.** The trial was conducted in a farrow-to-finish herd comprising 60 sows. For three months, all cohorts of nursery and finisher piglets within the herd were provided a diet supplemented with 0.1% MIIP from weaning to marketing. To evaluate PRRS immune responses, serum samples were randomly collected from five 8-week-old nursery pigs and five 20-week-old finisher pigs at the time of trial initiation and three months thereafter.

**Trial 3.** The trial was conducted in another farrow-to-finish herd comprising 60 sows. This herd had not received PRRS vaccination, though MIIP supplementation was implemented for one year before the study. Sixteen 4-week-old piglets from two litters, all PRRS antibody-negative, were randomly assigned to either the control group or the MIIP-treatment group. Two groups were kept in the next pen of the same house under the same field conditions. Piglets in the MIIP treatment group received a diet supplemented with 0.1% MIIP for one month. At eight weeks of age, serum samples were collected from all piglets for PRRS ELISA antibody analysis under natural PRRSV infection.

**Trial 4.** The field trial was conducted in a 400-sow farrow-to-finish herd. The farrowing facility operated under an all-in, all-out management system. The farm has a history of PRRS outbreaks, and the sows were vaccinated once every three months with PRRSV MLV vaccine (Ingelvac PRRSV MLV, Boehringer Ingelheim, St. Joseph, MO, USA). None of the piglets were administered the PRRS vaccine. During the trial period, the sows’ feed was supplemented with an oxytetracycline product at 200 ppm, and the creep feed contained colistin, halquinol, tilmicosin, and amoxicillin, which were administered to the piglets to manage bacterial infection. Before the trial, serum samples were collected from 10 randomly selected animals from each of the following groups: sows, 3-week-old piglets, and 6-week-old piglets in the herd. These samples were tested for PRRSV using qPCR and for PRRSV-specific antibodies using ELISA to assess the infection and immune status of the farm. Then, two batches of sows, separated by a one-week interval between their farrowing, were randomly allocated to the experimental or control group. This allocation resulted in 24 sows in the MIIP treatment group and 20 sows in the control group. For the MIIP treatment group, sows were fed MIIP brewer’s yeast at a daily dosage of 2 g from pregnancy at 80 days and then 4 g daily after farrowing. The piglets were weaned at 28 days of age. Prior to weaning, the piglets were fed a creep diet supplemented with 0.2% MIIP brewer’s yeast. After weaning, their nursery feed included 0.1% MIIP until they reached six weeks of age. After farrowing, serum samples were collected from 10 piglets in each group when the offspring reached 3 and 6 weeks of age, respectively. One piglet out of 10 sows was randomly selected from each group. Serum samples were collected from piglets and placed in Vacutainer^®^ Plus Plastic SSTTM tubes with polymer gel (BD Medical, East Rutherford, NJ, USA) for PRRSV antibodies and viral load determination. The milk of 5 sows from each group was also collected for viral neutralization assay and total IgA quantification at the end of the trial.

### 2.6. Statistical Analysis

Data regarding the PRRSV viral load, neutralization antibody titer, ELISA S/P ratios, and total IgA concentrations were expressed as the mean ± standard error (SE). Differences between groups were assessed using Student’s *t*-test for continuous variables. When continuous data exhibited clear outliers or deviated from a normal distribution, the Mann–Whitney U test (or Wilcoxon rank-sum test) was utilized as a nonparametric alternative. Categorical data, such as PRRSV positivity rates, were compared using Fisher’s exact test. A *p* value of less than 0.05 was considered statistically significant. All statistical analyses were performed using R version 4.0.3 (R Foundation for Statistical Computing, Vienna, Austria).

## 3. Results

### 3.1. Trial 1

**Figure 1:**
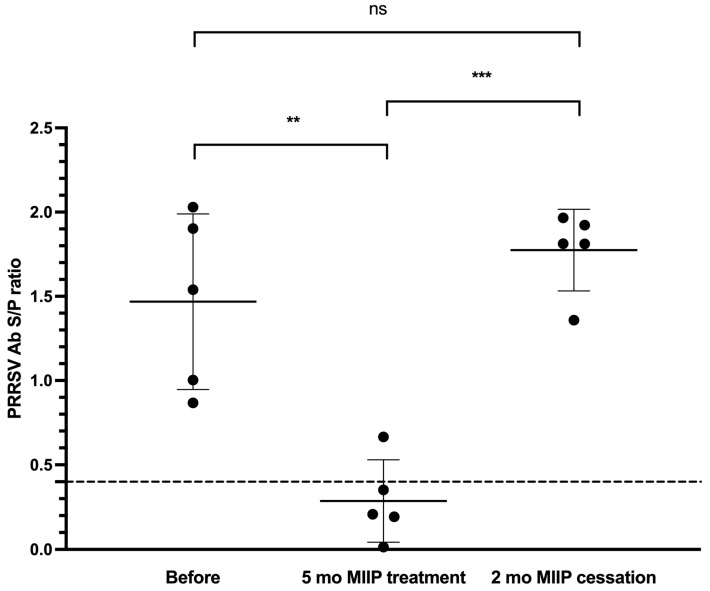
Dynamics of PRRSV-specific ELISA antibody titers in piglets before, during, and after MIIP yeast supplementation (Trial 1). The dotted line is the PRRSV ELISA interpretation threshold (0.4). ** and *** indicate significant differences between groups at *p* < 0.01 and *p* < 0.001, respectively.

Five months after administration of the MIIP treatment, a significant reduction in both PRRS antibody positivity and antibody titers was observed in piglets of the same age within the same herd (20% positivity; S/P ratio: 0.29 ± 0.24), compared to the values recorded five months earlier (100% positivity, S/P ratio: 1.47 ± 0.52) (*p* < 0.01). However, after the treatment was discontinued, antibody positivity rebounded to 100%, accompanied by a statistically significant increase in antibody titers (S/P ratio: 1.66 ± 0.26, *p* < 0.001) in piglets of the same age group two months post-discontinuation (Figure 1).

### 3.2. Trial 2

**Figure 2:**
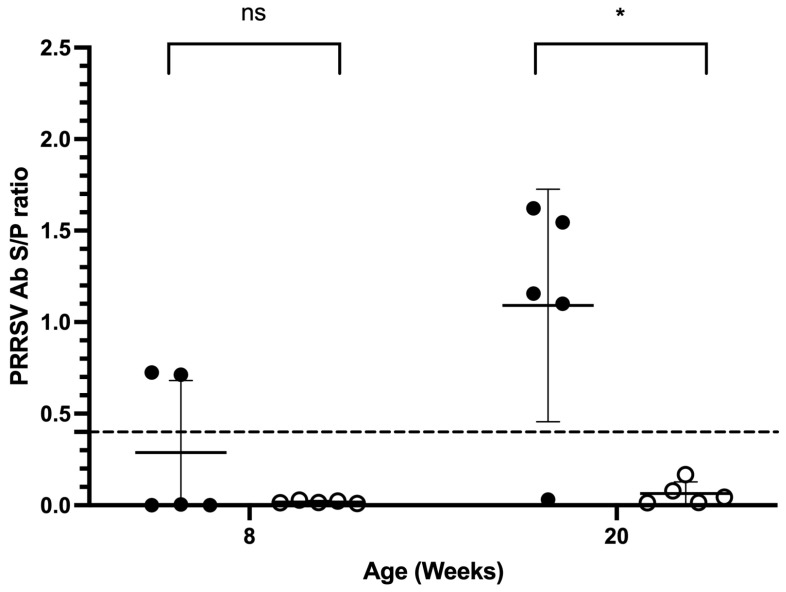
Effects of MIIP yeast supplementation on PRRSV-specific ELISA antibody titers in nursery (8 weeks old) and finisher (20 weeks old) pigs (Trial 2). Black and white circles represent before and after MIIP treatment, respectively. The dotted line is the PRRSV ELISA interpretation threshold (0.4). * indicates significant differences between groups (*p* < 0.05).

After a three-month MIIP supplementation period, eight-week-old piglets showed reductions in antibody positivity from 40% to 0% and in antibody titers from 0.288 ± 0.176 to 0.0182 ± 0.0034 (both *p* > 0.05). In 20-week-old pigs, antibody positivity declined from 80% to 0%, and titers decreased from 1.091 ± 0.284 to 0.064 ± 0.064, with both changes reaching statistical significance (*p* < 0.05) (Figure 2).

### 3.3. Trial 3

**Figure 3:**
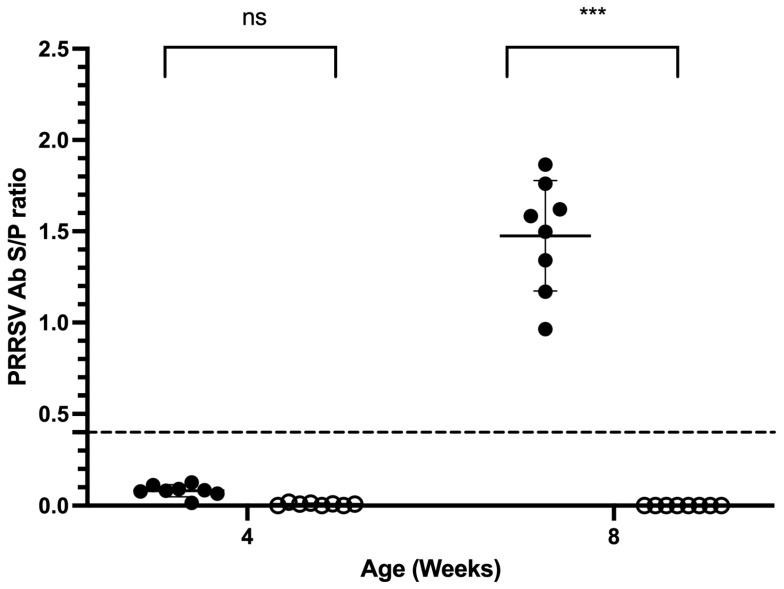
Impact of MIIP yeast supplementation on PRRSV-specific ELISA antibody titers in piglets from a PRRSV-positive herd (Trial 3). Black and white circles represent control and MIIP treatment groups, respectively. The dotted line is the PRRSV ELISA interpretation threshold (0.4). *** indicates significant differences between groups (*p* < 0.001).

At the onset of the study, all four-week-old piglets tested negative for PRRS antibodies, with a mean S/P ratio of 0.04 ± 0.04. After four weeks, the control group demonstrated a 100% seroconversion, with an S/P ratio of 1.48 ± 0.30 (*p* < 0.001). In contrast, the MIIP-treated group remained antibody-negative, exhibiting an S/P ratio of 0.00 ± 0.00 (*p* < 0.05), indicating a statistically significant difference between the two groups after MIIP treatment (Figure 3).

### 3.4. Trial 4

#### 3.4.1. Baseline PRRSV Status of the Study Farm Prior to the Trial

All ten sows tested seropositive for PRRSV antibodies according to the ELISA, with a mean S/P ratio of 1.07 ± 0.37. Quantitative PCR (qPCR) assays for both PRRSV-1 (European type) and PRRSV-2 (North American type) yielded negative results in all sows. In 3-week-old piglets, the ELISA seropositive rate was 80%, with an average S/P ratio of 0.78 ± 0.36. All piglets were qPCR-negative for PRRSV-1, while 40% were positive for PRRSV-2, with a mean viral load of 10^4.8^ copies/μL. At 6 weeks of age, 70% of piglets were ELISA seropositive (S/P = 0.76 ± 0.53), with continued negativity observed for PRRSV-1 qPCR. However, PRRSV-2 was detected in 70% of these piglets, with an average viral load of 10^3.48^ copies/μL.

#### 3.4.2. PRRSV qPCR Results

**Figure 4:**
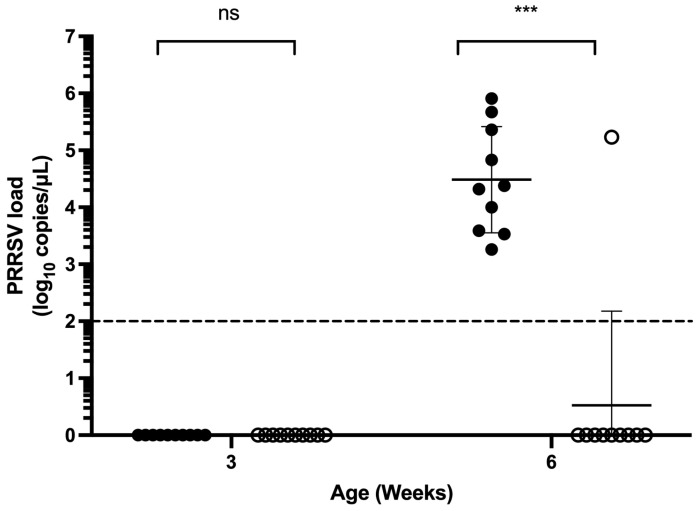
PRRSV-2 viral load in piglets fed diets with (white circle) or without (black circle) MIIP yeast supplementation at 3 and 6 weeks of age. The dotted line is the detection limit of PRRSV RT-qPCR. *** indicates significant differences between groups (*p* < 0.001).

The test results for Trial 4 are outlined in Table 1. Among the 3-week-old piglets in both MIIP and control groups, no PRRSV-1 or PRRSV-2 was detected by qPCR. At 6 weeks of age, all piglets in both groups remained negative for PRRSV-1. However, PRRSV-2 was detected in 10% of the piglets in the MIIP group, significantly lower than the 100% positivity observed in the control group (*p* < 0.001). The PRRSV-2 viral load in the single positive piglet from the test group (MIIP group) was 10^5.23^ copies/μL. In the control group, viral loads ranged from 10^3.26^ to 10^5.91^ copies/μL (Figure 4). Although the mean serum viral loads of the two groups did not differ significantly according to Student’s *t*-test (*p* > 0.05), the nonparametric Mann–Whitney U test (or Wilcoxon rank-sum test) revealed a significant difference (*p* < 0.001), suggesting a potential disparity in median viral loads.

#### 3.4.3. PRRSV ELISA Antibody Results

**Figure 5:**
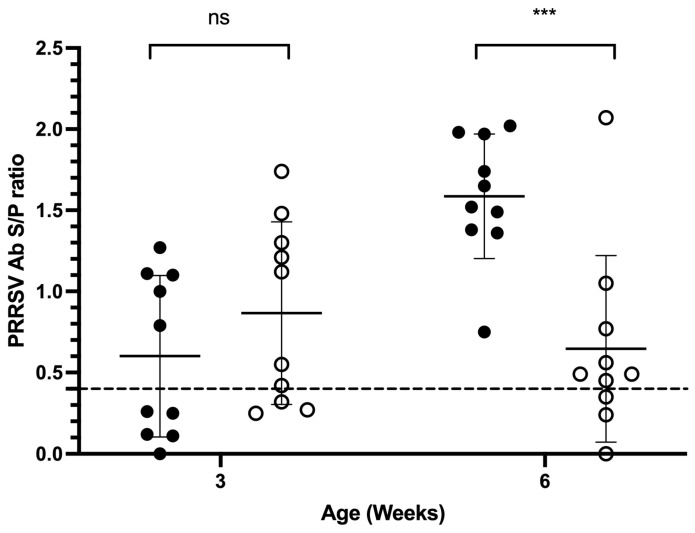
PRRSV-specific ELISA antibody titers in piglets fed diets with (white circle) or without (black circle) MIIP yeast supplementation at 3 and 6 weeks of age. The dotted line is the PRRSV ELISA interpretation threshold (0.4). *** indicates significant differences between groups (*p* < 0.001).

In 3-week-old piglets, the ELISA seropositive rate was 70% in the MIIP group (S/P = 0.87 ± 0.56) and 50% in the control group (S/P = 0.60 ± 0.50). No significant differences in either the seropositive rate or S/P ratio were observed between groups (*p* > 0.05; Figure 5). In 6-week-old piglets, the MIIP group maintained a 70% seropositive rate (S/P = 0.65 ± 0.58), while the control group reached 100% positivity and had a much higher level of antibody titers (S/P = 1.59 ± 0.38). Although the difference in positive rate was not statistically significant (*p* > 0.05), the control group exhibited a significantly higher level of antibody titers (*p* < 0.001; Figure 5).

#### 3.4.4. Total IgA Concentration and Neutralization Antibody

**Figure 6:**
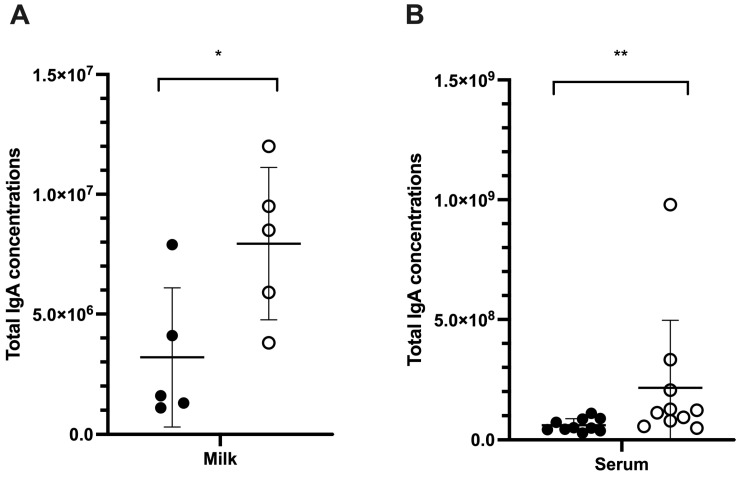
Total IgA concentrations in milk of sows (**A**) and in serum of piglets (**B**) fed diets with (white circle) or without (black circle) MIIP yeast supplementation (Trial 4). * and ** indicate significant differences between groups, *p* < 0.05 and *p* < 0.01, respectively.

Sows receiving MIIP supplementation had significantly higher milk IgA concentrations (7.94 × 10^6^ ± 3.18 × 10^6^ ng/mL) compared to the control group (3.2 × 10^6^ ± 2.89 × 10^6^ ng/mL; *p* < 0.05) (Figure 6A). At 6 weeks of age, the MIIP group exhibited a higher mean serum IgA concentration (2.16 × 10^8^ ± 2.81 × 10^8^ ng/mL) compared to the control group (0.61 × 10^8^ ± 0.26 × 10^8^ ng/mL) (*p* < 0.01) (Figure 6B). The mean level of PRRSV-neutralizing antibody titers in milk was higher in the MIIP group (64 ± 0.71) compared to the control group (27.86 ± 0.37), but the difference was not statistically significant (*p* > 0.05).

## 4. Discussion

This study investigated the immunomodulatory and antiviral efficacy of the *Saccharomyces cerevisiae* strain MIIP against porcine reproductive and respiratory syndrome virus (PRRSV) under commercial field conditions. Across all experimental trials, a notable reductions in PRRSV seropositivity and antibody titers were observed among piglets treated with MIIP yeast supplementation. In trials lacking a concurrent control (Trials 1 and 2), declines in PRRS ELISA seropositivity and antibody titers within the herd were evident after three or five months of MIIP yeast administration. A similar trend was observed in trials incorporating a concurrent control (Trials 3 and 4), further supporting the efficacy of the treatment against field conditions. This conclusion is supported by the corresponding decrease in viral load within the serum of piglets in the MIIP-treated group (Trial 4). Notably, in Trial 1, a resurgence in antibody positivity occurred following the cessation of MIIP treatment for two months, suggesting that the duration of supplementation is insufficient for sustained effects. Continued administration may be necessary to maintain antiviral benefits over time.

Pretrial assessments confirmed that the pig farm in Trial 4 was contaminated with PRRSV-2, and piglets typically became infected between three and six weeks of age, which is common in an infected environment. Piglets born from seropositive females usually contract the infection and seroconvert actively from the age of 3–6 weeks [6]. Subsequent results also indicated that all three-week-old piglets tested negative for PRRSV, while the PRRSV ELISA antibody positivity rate declined to 50–70%, suggesting the continued presence of maternal antibodies protecting against PRRSV infection. In contrast, when these piglets reached six weeks old, the ELISA antibody positivity rate of the control group increased from 50% at three weeks to 100%, and the mean value of antibody titers (S/P ratio) rose from 0.6 to 1.59. Given that the piglets had not been vaccinated against PRRSV and that 100% of the control group tested positive for PRRSV-2, these findings confirm the occurrence of PRRSV-2 infections between three and six weeks of age, as expected in an infected environment [6]. This time frame aligns with previous observations and is likely associated with the waning of maternal immunity. Conversely, in the MIIP group, only one 6-week-old piglet tested positive for the virus, a statistically highly significant difference compared to the 100% infection rate in the control group (*p* = 0.0001191). Although the *t*-test showed no statistically significant difference in the mean value of viral titers between the groups (*p* = 0.1839), this outcome may be attributable to the small sample size and the influence of outliers. Similar variability in immune responses to PRRSV across the swine population has also been observed [30,31]. Further analysis using the nonparametric Mann–Whitney U test (or Wilcoxon rank-sum test) revealed a significant difference (*p* = 0.00076), suggesting a potential disparity in median viral loads. The significantly lower rate of viremia positivity and reduced viral titers in the serum of the MIIP group strongly suggest that MIIP yeast exhibits antiviral efficacy.

The observed antiviral activity is likely attributable to the immunomodulatory properties of yeast cell wall components, particularly β-1,3-glucans and α-mannans, which interact with pattern recognition receptors (PRRs) on antigen-presenting cells such as macrophages and dendritic cells. This interaction is known to enhance cytokine signaling pathways, promote T-helper (Th) cell activation, and stimulate mucosal and systemic immune responses. Additionally, yeast supplementation has been reported to stabilize gut microbiota, thereby supporting tonic type I interferon (IFN-I) signaling and priming the host for antiviral immunity [32,33]. The lower amount of PRRSV-specific antibody titers observed in the MIIP group may reflect reduced viral antigen exposure due to lower infection rates, as shown in six-week-old piglets in trial 4. This phenomenon is more likely explained by reduced infection pressure rather than by suppressed humoral immunity. This reduction may be attributed to the immunomodulatory properties of *Saccharomyces* yeast, which are known to enhance antiviral defenses and potentially prevent PRRSV from entering the bloodstream via mucosal surfaces, leading to less systemic antigenic stimulation [27].

We also assessed total IgA levels as an indicator of mucosal immunity. Although differences in serum IgA concentrations did not reach statistical significance, the MIIP group consistently exhibited higher levels. Notably, total IgA levels in sow milk were significantly elevated in the MIIP group compared to controls (*p* = 0.037), suggesting enhanced maternal mucosal immunity, as suggested by Bi et al. [22]. While greater levels of PRRSV-neutralizing antibody titers in sow milk were observed in the MIIP group, the difference did not reach significance (Table 1), possibly due to field variability and the limited sample size. In Trial 4, antibiotics were administered to the herd to manage the bacterial outbreak. Since antibiotic-induced depletion of gut and lung microbiota has been linked to secondary IgA deficiency in intensive care unit patients [34], further investigation is warranted to determine whether comparable effects arise in pigs. Future studies should investigate the duration-dependent effects of MIIP supplementation and its potential synergy with PRRSV vaccination. They should also examine its role in modulating interferon signaling pathways, especially type I IFNs in the lungs, which are critical for controlling PRRSV replication [9]. Mechanistic studies employing immunohistochemistry, cytokine profiling, and transcriptomic analyses would also be valuable in elucidating the precise pathways involved. In addition, it would be interesting to evaluate animal health, reproductive indicators, and overall performance in pigs receiving the MIIP supplemental diet, thereby linking mechanistic insights with practical outcomes in swine production.

## 5. Conclusions

Collectively, these findings demonstrate that dietary supplementation with the *Saccharomyces cerevisiae* strain MIIP reduces PRRSV infection rates and enhances mucosal and systemic immunity in piglets. The significant reduction in viremia and antibody titers in treated groups suggests a promising role for MIIP as a non-antibiotic, immune-enhancing feed additive. Continued research into its immunological mechanisms and field application strategies could contribute meaningfully to PRRSV control and the development of PRRSV-free herds.

## Figures and Tables

**Table 1:** Immune responses and PRRSV level of pigs fed diets with or without dietary yeast *Saccharomyces cerevisiae*.

Age of Pigs	Tests	Group		*p* Value
		MIIP	Control	
3-week-old	PRRSV-1 qPCR Positive rate	0%	0%	-
	PRRSV-2 qPCR Positive rate	0%	0%	-
	PRRSV ELISA (S/P)/positive rate	0.87 ± 0.56 */70%	0.60 ± 0.50/50%	0.279 (*t*-test)/0.6499 (Fisher’s test)
6-week-old	PRRSV-1 qPCR Positive rate	0%	0%	-
	PRRSV-2 qPCR Positive rate	10%	100%	0.0001191 (Fisher’s test)
	PRRSV-2 qPCR Virus titers (copies/uL)	10^5.23^	10^5.21^ ± 10^4.93^	0.1839 (*t*-test), 0.00076 (Mann–Whitney U test or Wilcoxon rank-sum test)
	PRRSV ELISA (S/P)/positive rate	0.65 ± 0.58/70%	1.59 ± 0.38/100%	0.0005757 (*t*-test)/0.2105 (Fisher’s test)
	Total IgA ELISA in serum (ng/mL)	2.16 × 10^8^ ± 2.81 × 10^8^	0.61 × 10^8^ ± 0.26 × 10^8^	1.451 × 10^−8^ (*t*-test)
Sows	Total IgA ELISA in milk (ng/mL)	7.94 × 10^6^ ± 3.18 × 10^6^	3.2 × 10^6^ ± 2.89 × 10^6^	0.03721 (*t*-test)
	Mean PRRSV neutralizing antibody titers in milk (log2 titers)	64 ± 0.71	27.86 ± 0.37	0.1837 (*t*-test)

* mean ± se.

## Data Availability

The data presented in this study are available on request from the corresponding author due to ethical restrictions regarding participant confidentiality.
